# JQ1 synergizes with the Bcl-2 inhibitor ABT-263 against *MYCN*-amplified small cell lung cancer

**DOI:** 10.18632/oncotarget.21146

**Published:** 2017-09-21

**Authors:** Huogang Wang, Bo Hong, Xuemin Li, Ke Deng, Hong Li, Vivian Wai Yan Lui, Wenchu Lin

**Affiliations:** ^1^ High Magnetic Field Laboratory, Chinese Academy of Sciences, Hefei 230031, Anhui, P.R. China; ^2^ University of Science and Technology of China, Hefei 230036, Anhui, P.R. China; ^3^ Key Laboratory of High Magnetic Field and Ion Beam Physical Biology, Hefei Institutes of Physical Science, Chinese Academy of Sciences, Hefei 230031, Anhui, P.R. China; ^4^ School of Biomedical Sciences, Faculty of Medicine, The Chinese University of Hong Kong, Hong Kong SAR, P.R. China

**Keywords:** small cell lung cancer, N-Myc, Bcl-2, JQ1, ABT-263

## Abstract

Small cell lung cancer (SCLC) is a clinically aggressive cancer with very poor prognosis. Amplification of *MYC* family genes and overexpression of Bcl-2 protein are common in SCLC, and they are likely therapeutic targets for SCLC. Previous clinical study showed that single agent targeting Bcl-2 with ABT-263 was of limited efficacy in SCLC. In this study, we demonstrated for the first time that co-targeting of N-Myc and Bcl-2 resulted in marked synergistic antitumor effects in *MYCN*-amplified SCLC. We found that *MYCN*-amplified SCLC cells were highly sensitive to a Bromodomain and Extra-Terminal domain (BET) inhibitor JQ1, which was able to inhibit N-Myc protein expression. The inhibition of N-Myc by JQ1 induced the expression of Bim, and thereby sensitizing *MYCN*-amplified SCLC cells to ABT-263. The knockdown on Bim by siRNA reduced this JQ1/ABT-263 induced cell death. ABT-263 and JQ1 co-treatment in *MYCN*-amplified SCLC cells markedly disrupted Bim/Bcl-2 interaction, and prevented Bim’s interaction with Mcl-1. Importantly, this JQ1/ABT-263 co-targeting substantially inhibited the growth of *MYCN*-amplified SCLC xenografts *in vivo*. Our study demonstrates a new JQ-1/ABT-263 co-targeting strategy that can be employed for *MYCN*-amplified SCLC with high efficacy.

## INTRODUCTION

Small cell lung cancer (SCLC) is a clinically aggressive subtype of lung cancer, which accounts for 10∼15% of all lung cancers [1]. SCLC patients, who are diagnosed with extensive disease (i.e. spreading of cancer beyond the supraclavicular areas, or with distant metastasis) are treated with standard platinum-based chemotherapy plus etoposide. Although the initial response rate is quite high, almost all patients are bound to relapse within 3-6 months [2]. SCLC patients with extensive diseases have a median survival of less than 1 year [3]. Unlike non-small cell lung cancer (NSCLC), there is no targeted therapy being clinically approved for the treatment of SCLC.

The rare occurrences of druggable oncogenes or kinases make SCLC difficult to be harnessed for precision treatment. This is in contrast with non-small lung cancer (NSCLC), with which multiple druggable kinases are readily drugged in specific subgroups of NSCLC patients in a personalized manner. This includes Epidermal Growth Factor Receptor (EGFR) inhibitors for EGFR-mutated NSCLC and Anaplastic Lymphoma Kinase (ALK) inhibitors for ALK-rearranged NSCLC [4, 5]. In SCLC, the most frequently mutated genes are the non-druggable tumor suppressors, such as *TP53* and *RB1*[6]. Yet, amplification or overexpression of non-kinase oncogenes such as *MYC* family genes and Bcl-2 are very frequent events. In fact, Bcl-2 is over-expressed in 60%-90% of SCLC [7], while *MYC* family genes are amplified in 20%-30% of cases [6, 8]. Though these two obvious targets are common in SCLC, co-targeting of Bcl-2 and MYC pathway has not been explored previously.

Bcl-2 homology domains 3 (BH3) mimetics mimick the BH3 death domains and trigger apoptosis. They are an effective class of Bcl-2 inhibitor. A new generation of BH3 mimetic, ABT-263 directly binds Bcl-2 to block its interaction with Bim, thereby enabling Bim-mediated induction of apoptosis [9]. In a pan-cancer cell line study, ABT-263 has been shown to be more effective in inhibiting SCLC and hematologic malignancies, compared to other tumor types [7]. Further, a large scale drug screening study of Cancer Cell Line Encyclopedia (CCLE) also revealed a relatively lower IC_50_ values of ABT-263 in SCLC and hematologic cancer among other cancers [10].However, early phases of clinical trials showed that ABT-263 demonstrated unimpressive clinical activities in 26 SCLC patients, with only one patient having a partial response, while 9 patients had stable disease and 16 patients had disease progression [11]. These clinical findings revealed the limitation of ABT-263 monotherapy for SCLC.

Previous studies showed that cancer cells with high Bim/Mcl-1 ratios were sensitive to ABT-263 treatment [10]. Theoretically, the anti-tumor activity of ABT-263 can be enhanced by increasing Bim protein expression or reducing Mcl-1 expression. Recent study has shown that the target of rapamycin complex 1/2 (TORC1/2) inhibitor AZD8055, which reduces Mcl-1 protein level, is able to remarkably enhance antitumor activity of ABT-263 in SCLC [10]. But patients treated with TORC1/2 inhibitors were usually rash, mucositis, and fatigue. And based on previous experience, hyperglycemia was associated with mTOR inhibitors [12]. Therefore, we put forward a hypothesis that Bim induction by pharmacological approaches can potentially enhance ABT-263 activity in SCLC.

*MYC* family genes (*MYC*, *MYCN* and *MYCL*) are frequently amplified in SCLC [8]. JQ1, a Bromodomain and Extra-Terminal domain (BET) inhibitor can inhibit *MYC* transcription by disrupting the interaction of BET proteins and *MYC* promoter [13]. Furthermore, previous studies have demonstrated that *MYC* inactivation is able to increase Bim expression in lymphoma system [14]. Here, we hypothesized that targeting of *MYC* family oncogenes by JQ1 would induce Bim up-regulation in SCLC and results in sensitization to ABT-263. Our findings showed that *MYCN*-amplified SCLC cell lines were sensitive to JQ1. JQ1 induced Bim protein up-regulation, which sensitized *MYCN*-amplified SCLC cells to ABT-263. Co-treatment with ABT-263 and JQ1 effectively disrupted the interaction of Bim with Bcl-2 and prevented Bim’s interaction with Mcl-1. Further, *MYCN*-amplified SCLC xenografts are exquisitely sensitive to this JQ-1/ABT-263 combination. Our findings reveal a novel co-targeting strategy specifically for *MYCN*-amplified SCLC.

## RESULTS

### *MYCN*-amplified SCLC cell lines are sensitive to JQ1

**Figure 1:**
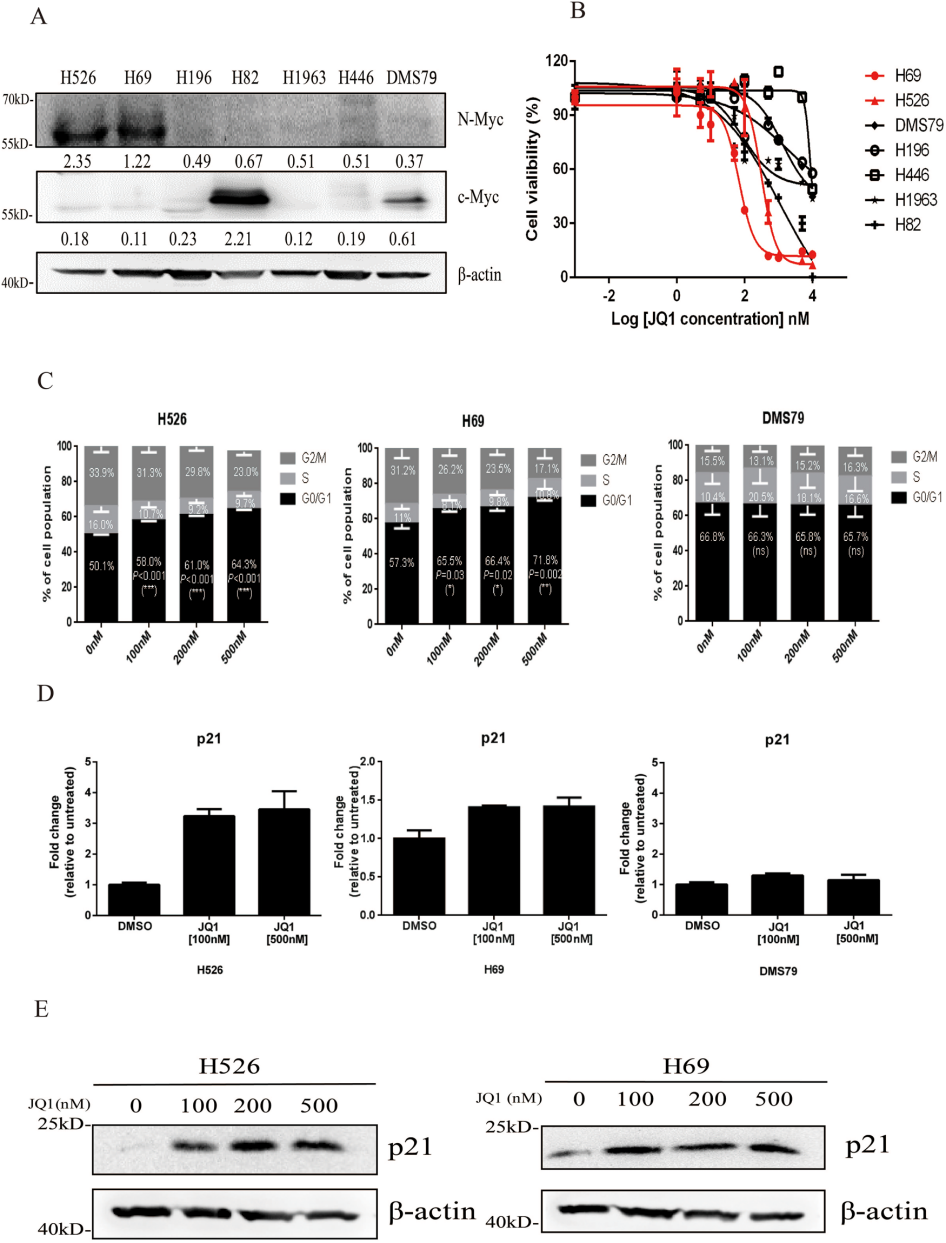
**(A)** Protein expression of c-Myc and N-Myc in a panel of SCLC cell lines by western blotting analysis. *β*-Actin was used as a loading control. Protein band intensities were quantified by ImageJ and normalized to Actin. **(B)** Growth inhibition curves of JQ1 in a panel of SCLC cell lines. SCLC cells were treated with different concentrations of JQ1 for 72 hours. CellTiter-Glo Luminescent assay was performed to evaluate the cell proliferation. Red curves represent *MYCN*-amplified cell lines, which are sensitive to JQ1. **(C)** Cell cycle distributions of H526, H69 and DMS79 cells treated with DMSO control or different concentrations of JQ1. Average percentage of cell population in different phases of cell cycle from 3 independent experiments was shown. Statistical significance of JQ1 treatment vs. DMSO control was indicated. **(D)** JQ1 induces p21 mRNA expression in *MYCN*-amplified SCLC cells. H526, H69 and DMS79 cells were treated with JQ1 or DMSO for 24 hours. Relative mRNA expression level of p21 was detected by quantitative RT-PCR. **(E)** JQ1 induces p21 protein expression in *MYCN*-amplified SCLC cells. H526 and H69 cells were treated with JQ1 or DMSO for 24 hours. Protein expression of p21 was detected by western blotting analysis. *β*-Actin was used as a loading control.

Nearly 20%-30% of SCLC tumors harbor amplification of *MYC* family genes [6]. We firstly detected the expression of c-Myc and N-Myc proteins in seven SCLC cell lines. As shown in Figure 1A, N-Myc was highly expressed in H526 and H69 cell lines, which have *MYCN* gene amplification [15] and c-Myc was highly expressed in H82 SCLC cell line that harbors *MYC* gene amplification [15] (Figure 1A). All 7 SCLC cell lines were then subjected to JQ1 treatment for 72 hours. As shown in Figure 1B and Table 1, we found that the two cell lines that have N-Myc protein overexpression, H526 and H69, were the most JQ1-sensitive lines with the lowest IC_50_ (236.1 nM and 667.1 nM). The c-Myc overexpressed H82 line was moderate sensitive to JQ1 (IC50: 1.01 μM), while the remaining 4 SCLC cell lines were the least sensitive ones with higher IC_50_ values of >8 μM). Our results indicate that *MYCN*-amplified SCLC cell lines are highly sensitive to JQ1.

**Table 1:** Cells sensitivities to JQ1 as well as basal levels of c-Myc and N-Myc expression in SCLC cell lines

	SCLC cell lines						
	High N-Myc		High c-Myc	Low c-Myc and N-Myc			
	H526	H69	H82	DMS79	H196	H1963	H446
JQ1 IC50 (nM)	236.1	667.1	1012	30383	26000	103755	8649
N-MYC expression	2.35	1.22	0.67	0.37	0.49	0.51	0.51
C-MYC expression	0.18	0.11	2.21	0.61	0.23	0.12	0.19

Previous studies have reported that JQ1 inhibits cancer cell growth through induction of cell cycle arrest in multiple cancer types, including leukemia, ovarian cancer and oral squamous cell carcinoma [16–19]. Therefore, we examined the effects of JQ1 on cell cycle progression in SCLC. As shown in Figure 1C and Supplementary Figure 1, in JQ1 sensitive SCLC cell line H526 and H69, JQ1 treatment significantly resulted in G_1_ arrest, with concomitant reduction in S phase population, when compared to DMSO control treatment. However, in JQ1 insensitive SCLC cell line DMS79, JQ1 did not induce any G_1_ changes. Since p21 protein is a key regulator of G_1_ checkpoint [20], we next examined if JQ1-induced G_1_ arrest was associated with p21 induction. As shown in Figure 1D, JQ1 treatment remarkably induced p21 mRNA expression in JQ1 sensitive cell line H526 and H69, but not in JQ1 insensitive cell line DMS79. Western blotting confirmed that p21 protein was induced by JQ1 treatment in H526 and H69 cells (Figure 1E). These results indicate that JQ1 induces growth inhibition of *MYCN*-amplified SCLC cells via G_1_ arrest.

### JQ1 increases Bim expression through suppressing N-Myc

**Figure 2:**
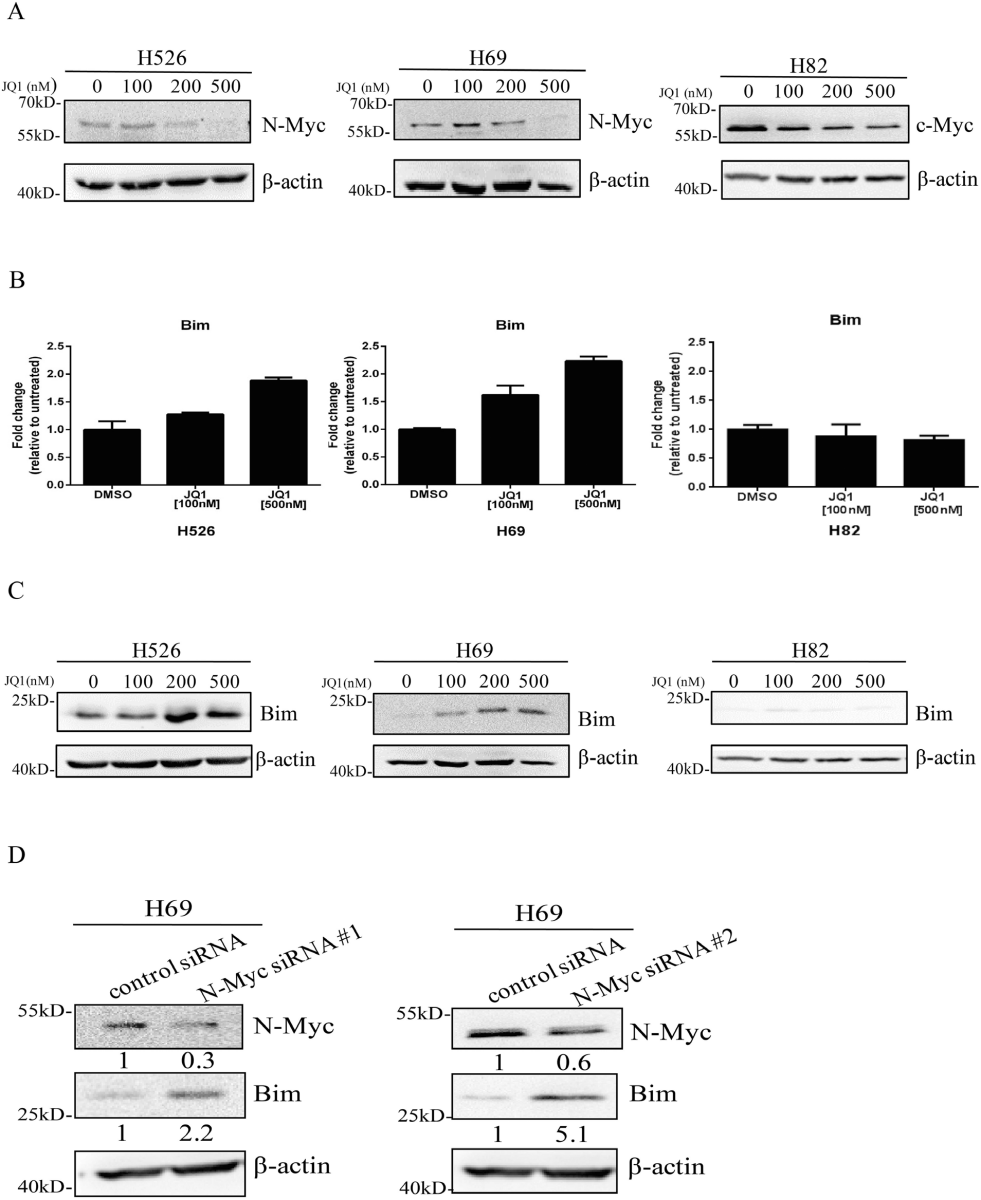
**(A)** JQ1 inhibits N-Myc and c-Myc expression in *MYCN* and *MYC*-amplified SCLC cells. H526, H69 and H82 cells were treated with JQ1 or DMSO for 24 hours. Protein expression of N-Myc and c-Myc was detected by western blotting. *β*-Actin was used as a loading control. **(B)** JQ1 induces mRNA expression of Bim in *MYCN*-amplified SCLC cells. H526, H69 and H82 cells were treated with JQ1 or DMSO for 24 hours. Relative mRNA level of Bim was detected by quantitative RT-PCR. **(C)** JQ1 induces protein expression of Bim in *MYCN*-amplified SCLC cells. H526, H69 and H82 cells were treated with JQ1 or DMSO for 24 hours. Protein expression of Bim was detected by western blotting. *β*-Actin was used as a loading control. **(D)** Knock-down of N-Myc increases Bim expression. H69 cells were treated with N-Myc siRNA #1, N-Myc siRNA #2 or their control siRNA for 48 hours, and then western blotting was performed to detect the expression levels of N-Myc and Bim. *β*-Actin was used as a loading control. The density of the bands was quantified by ImageJ and normalized to Actin.

Previous study has shown that JQ1 is able to reduce the expression of c-Myc in multiple myeloma models [17] and inhibition of c-Myc can induce Bim expression in lymphoma models [14]. Next, we determined if JQ1 was able to increase Bim expression through suppressing N-Myc and c-Myc in SCLC. As shown in Figure 2A, JQ1 decreased N-Myc and c-Myc expression in *MYCN*-amplified H526 and H69 cells as well as *MYC*-amplified H82 cells. Further, using quantitative RT-PCR, we confirmed that JQ1 treatment was able to induce Bim mRNA levels in a dose dependent manner in H526 and H69 cells (Figure 2B). This is further confirmed at the protein levels with western blotting that Bim protein expression was induced by JQ1 in both H526 and H69 cells (Figure 2C). On the contrary, in H82 cells, JQ1 treatment did not increase Bim expression in mRNA level, with only a very slight increase in Bim protein level (Figure 2B and 2C). In order to investigate whether Bim up-regulation was mediated via N-Myc down-regulation, we knocked down N-Myc expression with two N-Myc specific siRNAs #1 and #2. As shown in Figure 2D, knockdown of N-Myc increased Bim expression in H69 cells. These data demonstrate that JQ1 specifically increases Bim expression through suppression of N-Myc in *MYCN*-amplified SCLC.

### Bim up-regulation by JQ1 sensitizes *MYCN*-amplified SCLC cells to ABT-263

**Figure 3:**
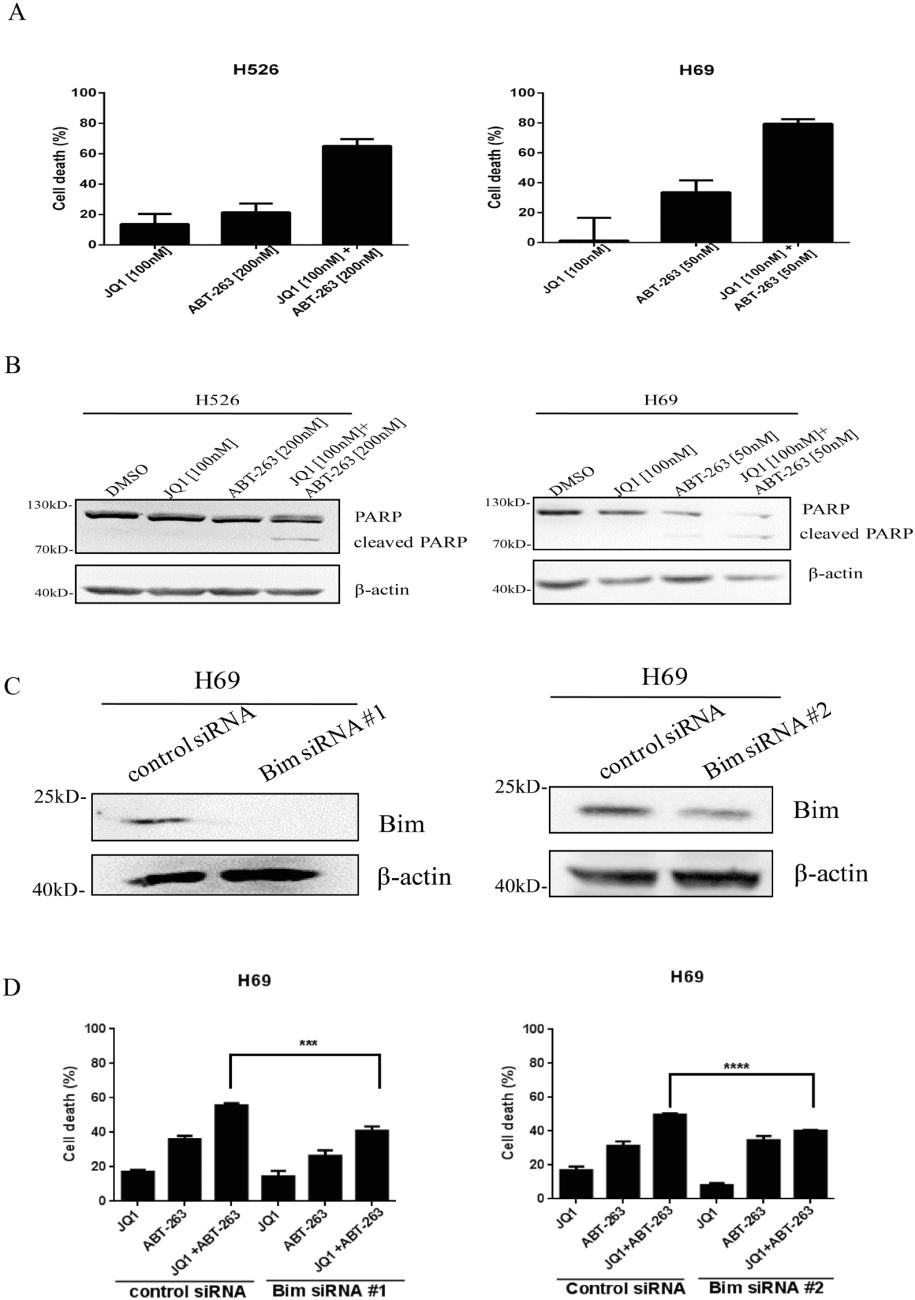
**(A)** Dramatical growth inhibition of *MYCN*-amplified SCLC cells induced by co-treatment of JQ1 and ABT-263. H526 and H69 cells were treated with DMSO control, JQ1, ABT-263 or the combination of JQ1 and ABT-263 for 72 hours. After treatment, growth inhibition was determined by CellTiter-Glo Luminescent assay. **(B)** JQ1 and ABT-263 co-treatment dramatically induces apoptosis in *MYCN*-amplified SCLC cells. H526 and H69 cells were treated with DMSO control, JQ1, ABT-263, or the combination of JQ1 and ABT-263 for 24 hours. PARP cleavage was detected by western blotting. *β*-Actin was used as a loading control. **(C)** Knock-down of Bim is confirmed by western blotting. After H69 cells were transfected by Bim siRNA #1, Bim siRNA #2 and control siRNA for 48 hours, cellular proteins were collected, and then Bim was detected by western blotting. **(D)** Knockdown of Bim significantly decreases the growth inhibition of H69 cells treated with combination of JQ1 and ABT-263. H69 cells were transiently transfected with Bim siRNA #1, Bim siRNA #2 and control siRNA for 24 hours. Then, transfected cells were plated in 96-well plate, and incubated with DMSO, JQ1 (100 nM), ABT-263 (50 nM) or the combination of JQ1 (100 nM) and ABT-263 (50 nM) for 72 hours. Cell proliferation was evaluated by CellTiter-Glo Luminescent assay.

As we hypothesized that Bim up-regulation by JQ1 sensitizes SCLC cells to ABT-263, we thereby examined whether combination treatment of JQ1 and ABT-263 exerted synergistic activity in inhibiting SCLC cell growth. We firstly tested the antitumor ability of ABT-263 in 7 SCLC cell lines. As shown in Supplementary Figure 2, SCLC cells exhibited different responses to ABT-263. DMS79, H69 and H1963 were sensitive to ABT-263 (IC_50_<1μM), while H526, H82, H196 and H446 were insensitive to ABT-263 (IC_50_>1μM). *MYCN*- or *MYC*-amplified H526, H69 and H82 cell lines were then subjected to JQ1/ABT-263 combination treatment. Our findings indicated that co-treatment of JQ1 and ABT-263 dramatically inhibited proliferation of *MYCN*-amplified H526 and H69 cell lines (Figure 3A), but not that of the *MYC*-amplified H82 cell line (Supplementary Figure 3). Furthermore, by calculating the combination index (CI) for synergism, we concluded that JQ1 and ABT-263 co-treatment exerted very strong synergistic anti-proliferative effects (CI<0.1) at all concentrations tested in H526 cells (Table 2). Next, we examined if this drug combination also enhanced apoptosis in *MYCN*-amplified H526 and H69 cells. We detected PARP cleavage (apoptotic marker) when H526 and H69 cells were treated with JQ1, ABT-263 or the combination. As shown in Figure 3B, the combination induced higher levels of cleaved PARP in H526 and H69 cells, when compared with either JQ1 or ABT-263 treatment alone.

**Table 2:** Combination index (CI) of JQ1 and ABT-263 in H526 cells

JQ1 dose (nM)	ABT-263 dose (nM)	Mean growth inhibition (%)	Dose of JQ1 alone with same inhibition (nM)	Dose of ABT-263 alone with same inhibition (nM)	CI
D1	D2	X	Dx1	Dx2	
50	10	63.9	575	2454	0.004
50	100	78.6	1621	5248	0.049
100	200	87.8	1698	6025	0.092
200	50	94.7	2238	8511	0.094
200	200	99.8	10964	11220	0.035

In order to determine whether JQ1 sensitize SCLC cells to ABT-263 was through Bim up-regulation, we knocked down Bim expression by two Bim siRNA #1 and #2. As shown in Figure 3C, Bim was effectively knocked down by both Bim siRNA treatment in H69 cells. Upon Bim knockdown, the inhibition of cell growth by JQ1 and ABT-263 co-treatment was significantly reduced in H69 cells (vs. control siRNA transfection) (Figure 3D). These results demonstrate that JQ1 sensitizes *MYCN*-amplified SCLC cells to ABT-263 via Bim up-regulation.

### Combination of JQ1 and ABT-263 disrupts the interaction of Bim/Bcl-2 and prevents Bim’s interaction with Mcl-1 in *MYCN*-amplifed SCLC cells

**Figure 4:**
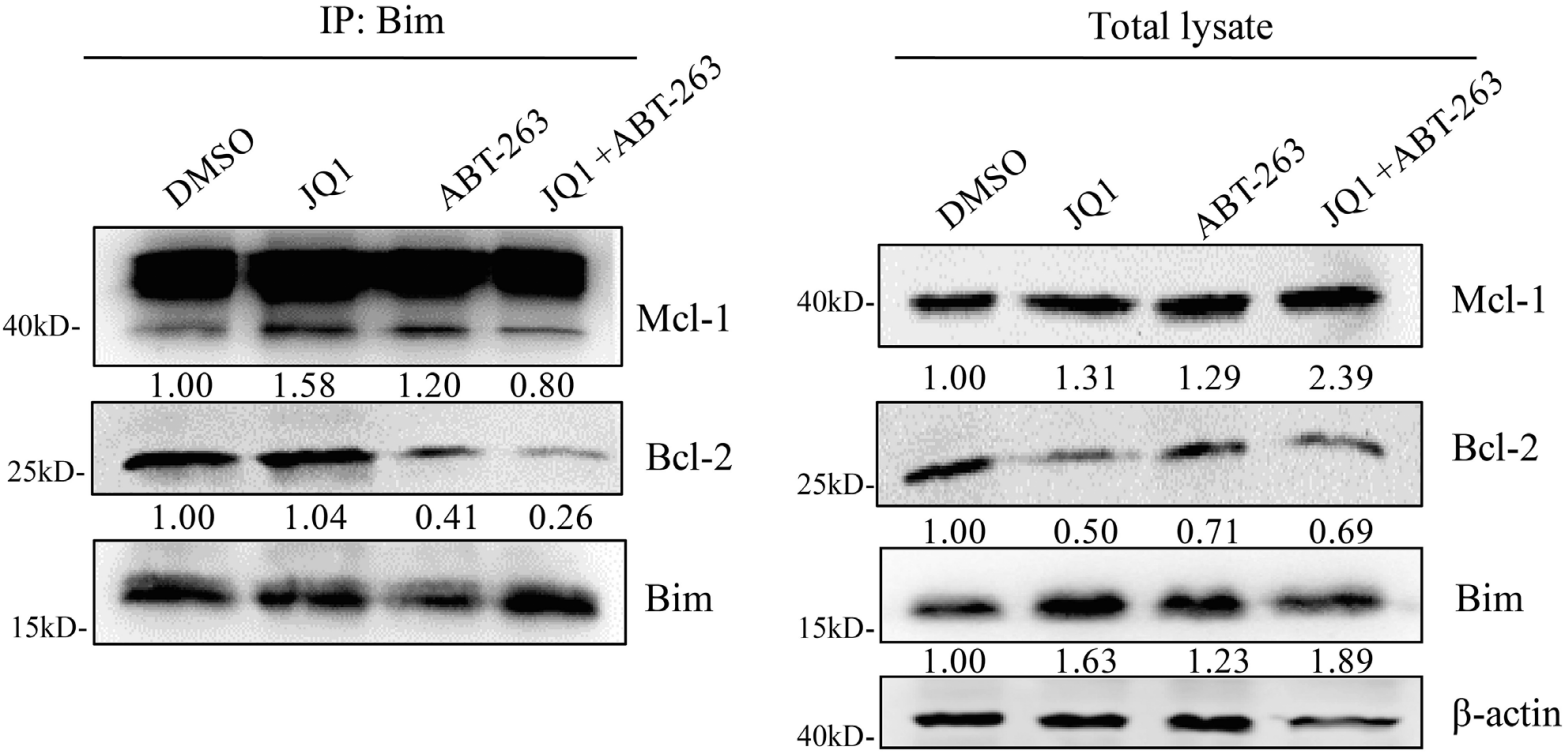
Combination treatment of JQ1 and ABT-263 strongly disrupts the interaction of Bim with Bcl-2 and Mcl-1 H526 cells were treated with DMSO control, JQ1 (100 nM), ABT-263 (200 nM), JQ1 (100 nM) plus ABT-263 (200 nM) for 24 hours. After treatment, Cells were lysed in immunoprecipitation lysis buffer. Cellular extracts were immunoprecipitated with 1 μg anti-Bim antibody. Precipitates were analyzed by western blotting to detect Bim, Mcl-1 and Bcl-2 proteins. The total lysates were also subjected to western blotting to detect the expression of Bim, Mcl-1 and Bcl-2 proteins. *β*-Actin was used as a loading control. Protein band intensities were quantified by ImageJ.

Anti-apoptotic proteins Bcl-2 and Mcl-1 can block apoptosis by interacting with the pro-apoptotic protein Bim. Previously, ABT-263 has been shown to block the binding of Bcl-2 to Bim, but does not block the binding of Mcl-1 to Bim [10]. To indicate whether combination treatment of JQ1 and ABT-263 is able to disrupt Bcl-2/Bim or Mcl-1/Bim interaction, we performed immunoprecipitation assay. As shown in Figure 4, ABT-263 treatment alone disrupted the interaction of Bim and Bcl-2. Surprisingly, we found that an even more substantial disruption of the Bcl-2/Bim complex was observed with the JQ1/ABT-263 combination treatment. Consistent with previous study [10], we found that ABT-263 treatment disrupted the interaction of Bim with Bcl-2, but the released Bim from Bcl-2/Bim complex was captured by Mcl-1 (Figure 4). Interestingly, combination treatment of JQ1 and ABT-263 prevented the released Bim from binding to Mcl-1. Our data suggest that the interaction of Bim with Bcl-2 or Mcl-1 is strongly disturbed by JQ1 and ABT-263 co-treatment, resulting in more Bim to induce apoptosis.

### JQ1/ABT-263 combination substantially inhibits SCLC tumor growth *in vivo*

**Figure 5:**
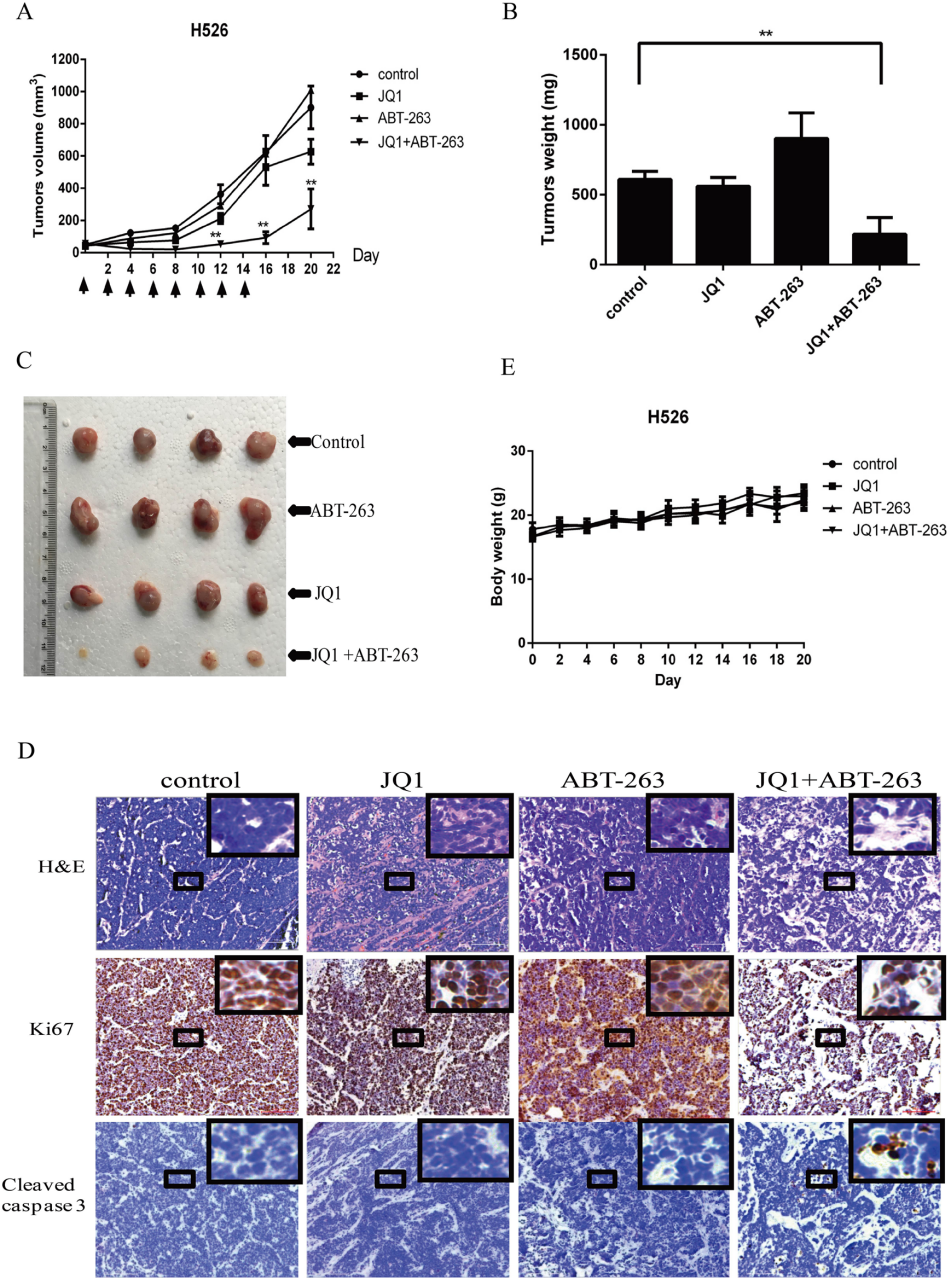
**(A)** Combination treatment with JQ1 and ABT-263 leads to robust antitumor activity in *MYCN*-amplified SCLC xenograft. H526 xenografts were treated by DMSO control, 25 mg/kg JQ1, 80 mg/kg ABT-263, or the combination of JQ1 (25 mg/kg) and ABT-263 (80 mg/kg) every two days for 2 weeks. The mean tumor size ± SEM is shown (**, P < 0.01 by an unpaired t test). **(B)** Tumor weights in JQ1+ABT-263 group were significantly decreased compared to control group (**, p < 0.01, by an unpaired t test). Data of tumor weights are expressed as means ±SEM. **(C)** Imaging of representative tumors from each group. The tumors were excised at the end of the experiment. **(D)** Histologic analysis of tumors, including H&E staining, immunohistochemical detection of Ki67 or cleaved Caspase 3. Scale bars represent 100 μm. Small boxed-areas were enlarged. **(E)**. There are no obviously difference in mouse weights from H526 xenografts during the course of treatment, comparing to control group. Detection was conducted in every two days from day 0 to the end of experiment. The mean body weight ± SEM is shown.

To evaluate the synergistic antitumor activity of JQ1 and ABT-263 *in vivo*, we established a human tumor xenograft model by subcutaneously injecting *MYCN*-amplified H526 SCLC cells into nude mice. Tumor-bearing mice were intraperitoneally treated with vehicle control, JQ1, ABT-263, or the combination of JQ1 and ABT-263 every two days for 14 days. As shown in Figure 5A, 5B and 5C, combination treatment with JQ1 and ABT-263 significantly inhibited tumor growth of H526 xenografts as compared with vehicle-treated control, but treatment with JQ1 or ABT-263 alone did not inhibit tumor growth. H&E staining of tumor sections revealed that combination treatment of JQ1 and ABT-263 decreased tumor cell density (Figure 5D). Ki67 expression was found to be strongly decreased in tumors treated by JQ1 plus ABT-263, whereas apoptosis (cleaved Caspase 3 staining) was increased in the tumors treated with the JQ1/ABT-263 combination (Figure 5D). No significant difference in body weight was observed among vehicle control, JQ1, ABT-263 or the combination group (Figure 5E). The results indicate that combination treatment of JQ1 and ABT-263 significantly inhibits tumor growth in *MYCN*-amplified SCLC xenografts.

## DISCUSSION

Currently, no targeted therapy is available for SCLC due to the lack of therapeutically relevant genome alterations. *MYC* family oncogene (MYC, *MYCN* or *MYCL*) is amplified in 20%-30% of the SCLC patients, thereby representing a potential therapeutic target in SCLC [6]. However, *MYC* family oncogene, as a non-kinase oncogene, is a challenging target for drug development. BET domain proteins act as epigenetic factors associated with acetylated histones and facilitate transcription of target genes, including *MYC*, *MYCN* and *MYCL*[17, 21, 22]. Recently, several groups reported that indirectly targeting *MYC* by the BET bromodomain inhibitor JQ1 exhibited anti-tumor activity in childhood sarcoma, thyroid tumor and endometrial cancer [23–25]. In SCLC, previous studies indicated that JQ1 targeted *MYCL* and *ASCL1* to inhibit cancer cell growth [26, 27]. In this study, we demonstrated that JQ1 was able to down-regulate *MYCN* gene encoding protein N-Myc, leading to growth inhibition of *MYCN*-amplified SCLC cells. Interestingly, our study found that *MYCN*-amplified SCLC cells were the most sensitive to JQ1, which agree with a previous report by Baratta et al. indicating that JQ1 sensitivity strongly correlated with *MYCN* expression levels [28]. Thus, our study demonstrates that JQ1 is more effective in *MYCN*-amplified SCLC cells.

In this study, we found that JQ1 induced Bim in SCLC, which is consistent with recently several reports indicating that JQ1 is able to up-regulate Bim in hepatocellular carcinoma and leukemia [29, 30]. Furthermore, our study found that Bim induction by JQ1 is through N-Myc inhibition, as N-Myc inhibition by siRNA treatment up-regulated Bim. To our knowledge, our study is the first report to indicate that N-Myc inhibition is able to up-regulate Bim expression. Previous study has demonstrated that c-MYC inactivation up-regulated Bim through suppressing miR-17-92, a key post-transcriptional repressor of Bim in *MYC*-induced lymphomas model [14]. However, in SCLC, our study found that c-Myc inhibition did not up-regulate Bim expression by unknown reason. Further study needs to investigate how N-Myc inhibition up-regulates Bim, and why c-Myc inhibition does not up-regulate Bim in SCLC.

Previous study has indicated that cancer cells with higher Bim and lower Mcl-1 expression is more sensitivity to ABT-263. The down-regulation of Mcl-1 by mTOR inhibitor AZD8055 sensitizes SCLC cells to ABT-263 [10]. Ham et al. also reported that Arora Kinase inhibitor MLN8237 sensitized *MYCN*-amplified neuroblestoma to BH3 mimics ABT-199 partially via Mcl-1 reduction [31]. In our study, we found that JQ1 up-regulated Bim, but had no impact on Mcl-1 expression (Supplementary Figure 4). Furthermore, we indicated that Bim up-regulation by JQ1 is able to sensitize *MYCN*-amplified SCLC cells to ABT-263. In contrast, JQ1 did not induce Bim in *MYC*-amplified H82 cells, and thereby JQ1 did not sensitize H82 cells to ABT-263. In addition, we found that Bcl-2 expression was lower in H82 cells than that in H526 and H69 cells (Supplementary Figure 5), which may also be a reason why no synergistic effect of JQ1 combined with ABT-263 was observed in H82 cells. Our study suggests that the elevation of Bim level by pharmacological treatment may be an effective strategy to sensitize cancer cells to ABT-263.

The proapoptotic activity of Bim is regulated by the interaction of Bim with anti-apoptotic protein Bcl-2 and Mcl-1. Our immunoprecipitation data of Bim showed that ABT-263 treatment disrupted the interaction of Bim with Bcl-2, whereas the released Bim bound to Mcl-1. These results are consistent with previous study by Faber et al.[10], indicating that Mcl-1 binds to the Bim that is released from Bim/Bcl-2 complex upon ABT-263 treatment. Therefore, the inability of disrupting the interaction of Bim with Mcl-1 limits the activity of ABT-263. Notably, our study found that combination treatment of ABT-263 and JQ1 not only led to more release of Bim from the Bim/Bcl-2 complex, but also prevented the released Bim binding to Mcl-1. Taken together, our study suggests that combination of JQ1 and ABT-263disrupts the interaction of Bim/Bcl-2 and prevents the interaction of the released Bim with Mcl-1, which may promote strong apoptotic response.

**Figure 6:**
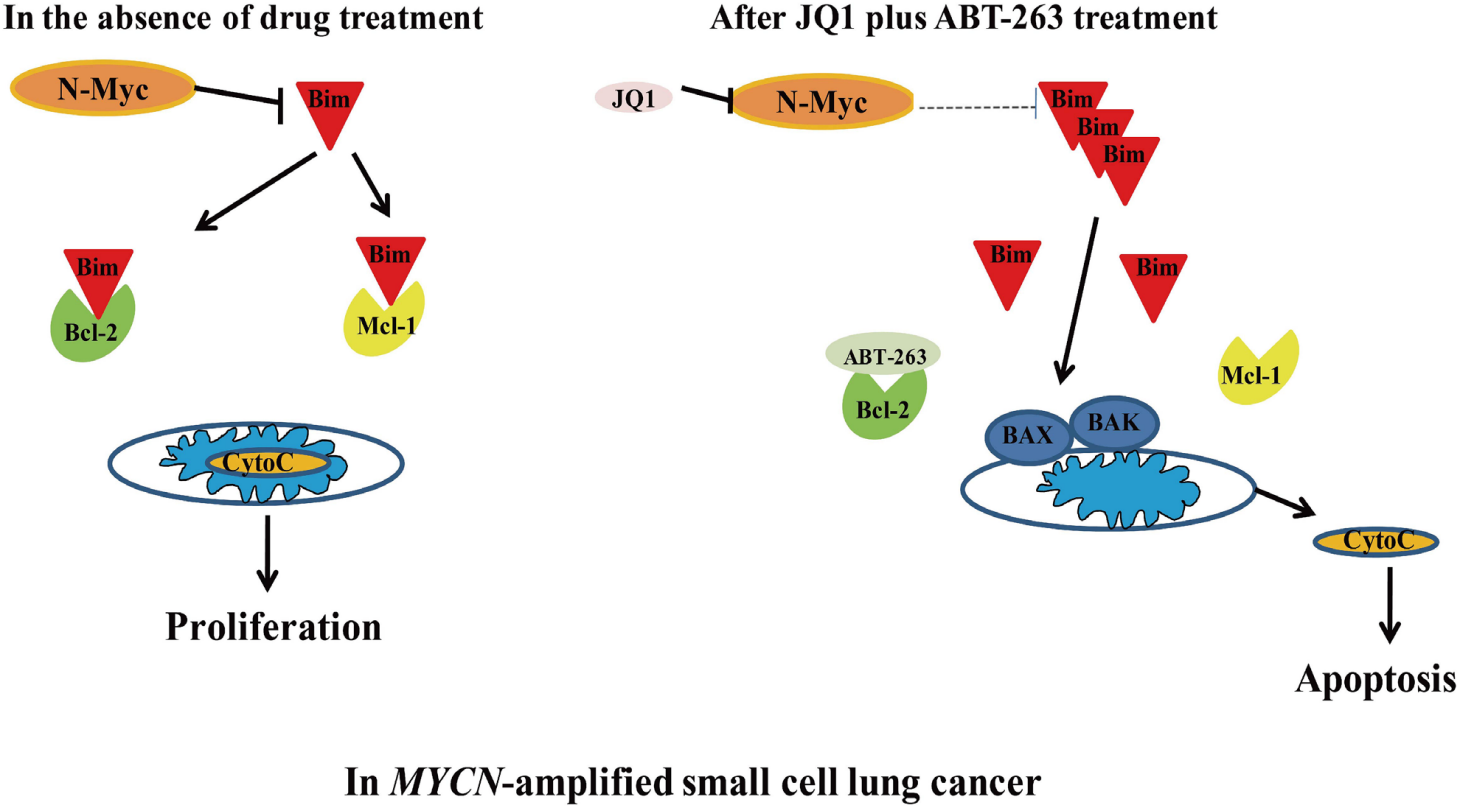
Schematic of *MYCN*-amplified SCLC before and after treatment with JQ1/ABT-263 combination In *MYCN*-amplified SCLC, N-Myc inhibits Bim. JQ1 treatment decreases N-Myc to up-regulate Bim. Combination of JQ1 with ABT-263 disrupts Bim’s interaction with BCL-2 and Mcl-1, leading to the liberation of Bim and apoptosis.

In summary, our study demonstrates that targeting N-Myc by JQ1 sensitizes *MYCN*-amplified SCLC cells to ABT-263. The synergistic anti-tumor activity of JQ1 and ABT-263 is through up-regulation of Bim, and disturbing the interaction of Bim with Bcl-2 and Mcl-1 (Figure 6). Furthermore, our *in vivo* experiment demonstrated that the combination of JQ1 and ABT-263 leads to marked tumor regression in *MYCN*-amplfied SCLC xenografts. In clinical medicine, patients with *MYCN*-amplified SCLC have been associated with tumor aggressiveness and shorter survival [32]. Altogether, our study reveals for the first time that co-targeting of N-Myc and Bcl-2 by JQ1/ABT-263 combination is a novel and effective strategy specifically for *MYCN*-amplified SCLC.

## MATERIALS AND METHODS

### Materials

JQ1 and ABT-263 were purchased from Selleck chemical (Shanghai, China) and stock solutions were prepared in DMSO (Sigma–Aldrich, Saint Louis, MO, USA) at a concentration of 10 mM. Antibodies against p21, Bcl-2, N-Myc, c-Myc, Bim, PARP, cleaved-Caspase 3 and Mcl-1 were from Cell Signaling Technology, Danvers, MA, USA. Ki67 antibody was from ZSGB-BIO, Beijing, China. Actin antibody was from TransBionovo, Beijing, China. The siRNA #1 and #2 against human Bim as well as its negative control siRNA were purchased from Cell Signaling Technology (Danvers, MA, USA). And the siRNA #1 against human N-Myc as well as its negative control siRNA were purchased from Santa Cruz Biotechnology, Dallas, Texas, USA. The siRNA #2 against human N-Myc as well as its negative control siRNA were pruchased from OriGene Technologies, Rockville, MD, USA.

### Cell lines

The human SCLC cell lines, H82, H526, DMS79, H69, H1963, H446 and H196 were maintained in RPMI-1640 media supplemented with 10% fetal bovine serum and 1% penicillin/streptomycin in a humidified incubator at 37°C in 5% CO2. All of these SCLC cell lines were kindly provided by Dr. Matthew Meyerson at Dana-Farber Cancer Institute, USA. RPMI-1640 media, FBS and penicillin/streptomycin were purchased from Gibco, Life Technologies, Carlsbad, CA, USA.

### Cell viability assay

SCLC cells were treated with DMSO control or drugs for 72 hours, and then cell viability was measured by using CellTiter-Glo Luminescent assay (Promega, Madison, WI, USA) according to the manufacturer’s instructions. Luminescence was measured in a multi-label plate reader (Envision PerkinElmer, USA). Data were normalized to DMSO control and represented by the mean of three independent measurements with SEM. The IC_50_ values were determined from the sigmoidal dose–response curves using PRISM4 software (GraphPad Software, Inc., La Jolla, CA, USA).

### Cell cycle analysis

After treatment with DMSO or JQ1 for 24 hours, cells were fixed by dropwise addition of ice-cold EtOH. Fixed cells were stained with PI/RNase staining buffer (BD Biosciences, Franklin, NJ, USA), and then analyzed by a FACS Calibur (BD Biosciences, Franklin, NJ, USA). The proportion of cells in each cell cycle phase was determined using ModFit software (Verity Software House, Topsham, ME, USA).

### Western blotting

After drug treatment, cells were homogenized in lysis buffer (150mM NaCl, 50mM Tris-HCl pH 8.0, 1% Triton X-100, and 1mM EDTA). Protein extracts were quantified by BCA Protein Assay Kit (Beyotime, Shanghai, China), separated by SDS-PAGE, and then transferred onto nitrocellulose blotting membrane (Cell Signaling Technology, Danvers, MA, USA). The membranes were hybridized with a primary antibody at 4 °C overnight followed by incubation with a secondary antibody for 2 hours at room temperature. Signals were visualized using SuperSignal West Pico chemiluminescent substrate (Thermo Scientific, Rockford, IL, USA).

### Quantitative RT-PCR

Total RNA was isolated using Trizol (Thermo Scientific, Rockford, IL, USA) together with Qiagen RNeasy Mini Kit (Qiagene, Hilden, Germany). cDNA was synthesized by Transcriptor First Strand cDNA Synthesis Kit (Roche, Mannheim, Germany) according to the manufacturer’s instructions. The quantitative real-time PCR was performed in triplicate using a FastStart Essential DNA Green Master (Roche, Mannheim, Germany) on a Roche LightCycler 96 Real Time PCR System. The expression levels of all tested genes were normalized to the expression level of β-actin. The cDNA was amplified with the following primers.

Bim
forward 5′-CCCCGCTTTTCATCTTTATG-3′,
reverse5′-GGGCTCCTGTCTGTGTCAA-3′
p21
forward 5′-GGCAGACCAGCATGACAGATT-3′,
reverse 5′-GCGGATTAGGGCTTCCTCT-3′
β-actin
forward 5′-CATGTACGTTGCTATCCAGGC-3′,
reverse 5′-CTCCTTAATGTCACGCACGAT-3′

### Immunoprecipitation (IP)

After treatment, cells were lysed with immunoprecipitation lysis buffer (250mM NaCl, 50mM Tris-HCl pH7.4, 0.5% Triton X-100, 10% glycerol). Five hundred micrograms of whole-cell extracts were incubated with 1 μg of Bim antibody followed by rocking at 4°C for overnight. After incubation, 50 μL of protein A Megnetic beads (Thermo Scientific, Rockford, IL, USA) were added, and then the samples were rocked at 4°C for 4 hours. After washed three times, samples were resuspended in 20 μL of 2 X sample buffer, and boiled for 10 minutes, and followed by SDS-PAGE.

### siRNA treatment

For Bim siRNA transfection, H69 cells (2 x10^5^) were seeded onto 6-well plates and incubated for 24 hours. Cells were then transfected with Bim siRNA or control siRNA using Effectene Transfection Reagent (QIAGEN, Hilden, Germany). Two days after transfection, one part of transfectants was collected for western blot to check whether Bim was knocked down. Other part of transfectants was replated onto 96-well plate and was treated with DMSO or drugs for 72 hours, and then cell viability was measured by CellTiter-Glo Luminescent assay.

For N-Myc siRNA transfection, H69 cells (2 x10^5^) were seeded onto 6-well plates and incubated for 24 hours. Cells were then transfected with N-Myc siRNA or control siRNA using Effectene Transfection Reagent (QIAGEN, Hilden, Germany). Two days after transfection, cell lysis was collected for western blotting.

### Xenograft experiments

Animal experiments were carried out according to a protocol approved by Institutional Animal Care and Use Committee of Hefei Institutes of Physical Science. Athymic nude mice were injected subcutaneously in dorsal flank, with a 100 μL suspension of 2 x 10^6^ H526 cells in an equal volume of Matrigel (BD Biosciences, Franklin, NJ, USA). When tumors grew to 4 to 5 mm in diameter, the mice were treated by intraperitonel injection with DMSO control, JQ1 (25 mg/kg), ABT-263 (80 mg/kg) and the combination of JQ1 (25 mg/kg) with ABT-263 (80 mg/kg) every 2 days. The tumor size was monitored by caliper measurements and calculated by the formula: Volume=(length×width×width)/2.

### H&E staining

Tumors were harvested from euthanized mice, fixed in 4% paraformaldehyde for 24 hours and embedded in paraffin wax. After sections were cut in 6 μm, sections were dewaxed, rehydrated and dipped into Mayer’s hematoxylin with agitation for 3-5 min. After rinsed by H_2_O, sections were stained with 1% eosin Y solution with agitation for 1-3 min. Subsequently, the sections were dehydrated with alcohol and xylene. Finally, mounting medium was added prior to covering with a cover slip.

### Immunohistochemistry

Tissues were cut as 6 μm sections. After dewaxed and reydrated, sections were placed in citrate buffer (0.01 M, pH 6.0) antigen retrieval solution boiled for 3 min in a pressure cooker. After blocked with 5% BSA, sections were incubated with Ki-67 antibody or cleaved-Caspase 3 antibody at 4°C overnight. Then, sections were incubated with secondary antibody and stained with DAB (ZSGB-BIO, Beijing, China). Finally, sections were counterstained with hematoxylin, and dehydrated, and then mounted.

### Assessment of drug synergy

Drug synergy was determined quantitatively using the combination index (CI) method of Chou and Talalay [33]. CI was calculated using the formula: CI=D_1_/DX_1_+D_2_/DX_2_, in which D_1_ and D_2_ are the doses used to achieve a specific growth inhibition when two drugs combined, and DX_1_ and DX_2_ are individual drug doses needed to achieve similar growth inhibition. CI<1 indicates synergism, whereas CI>1 indicates antagonism.

### Statistical analysis

All data were analyzed using PRISM4 Software (GraphPad Software, Inc., La Jolla, CA, USA). Statistical analysis was performed using an unpaired *t*-test. Results were considered as statistically significant when *P*< 0.05.

## SUPPLEMENTARY MATERIALS FIGURES



## Abbreviations

(SCLC): Small cell lung cancer
(CCLE): Cancer Cell Line Encyclopedia
(CI): Combination index
(NSCLC): Non-small cell lung cancer
(EGFR): Epidermal Growth Factor Receptor
(ALK): Anaplastic Lymphoma Kinase
(BH3): Bcl-2 homology domains 3
(BET): Bromodomain and Extra-Terminal
(TORC1/2): Target of rapamycin complex 1/2
