# Supplementary material for: JQ1 synergizes with the Bcl-2 inhibitor ABT-263 against MYCN-amplified small cell lung cancer
Source: Oncotarget. 2017 Sep 21;8(49):86312–24. doi: 10.18632/oncotarget.21146 (PMC5689687; doi:10.18632/oncotarget.21146)
Supplement: Supplementary file 1 [file oncotarget-08-86312-s001.pdf]

JQ1 synergizes with the Bcl-2 inhibitor ABT-263 against *MYCN*-amplified small cell lung cancer

SUPPLEMENTARY MATERIALS

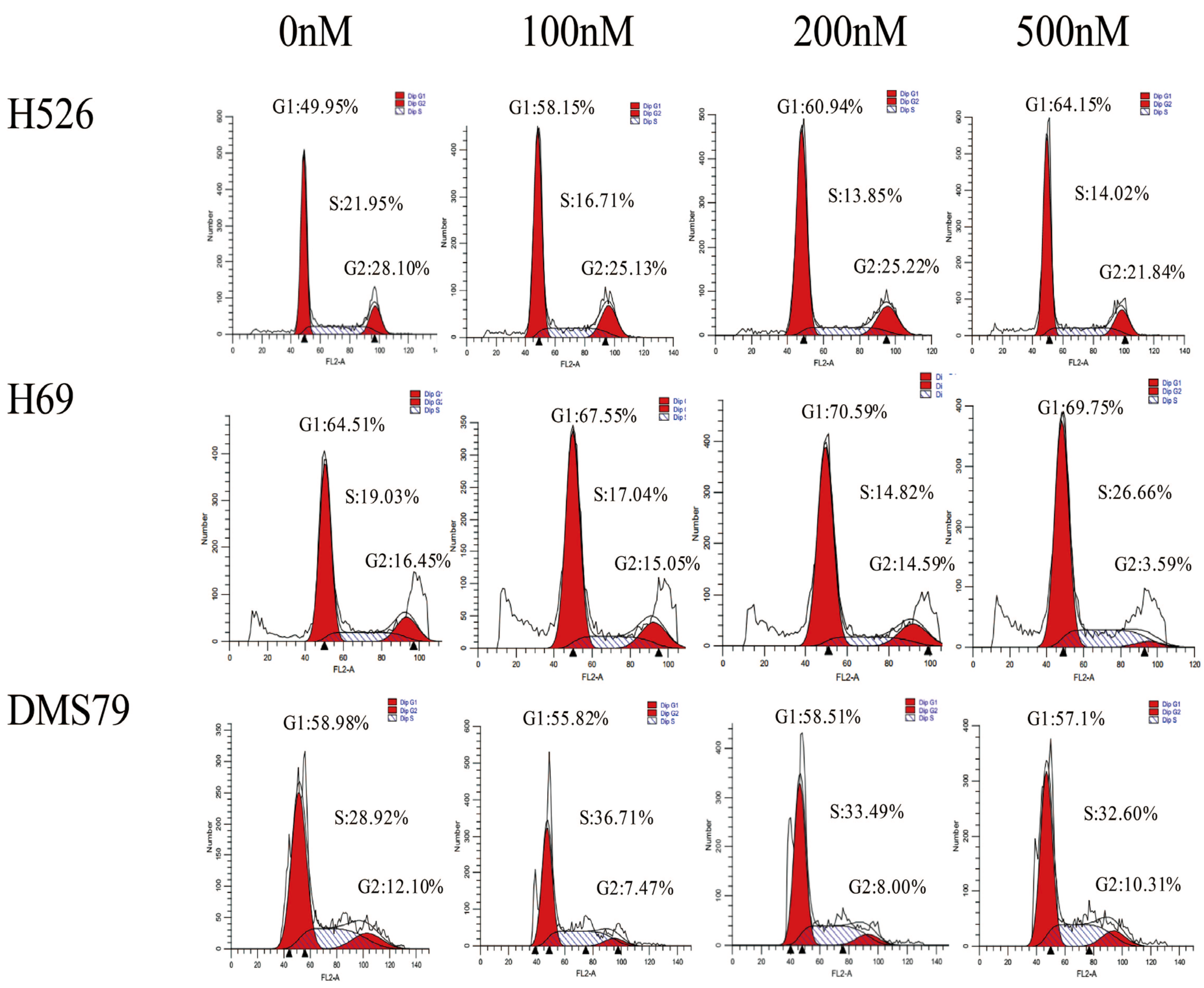

Supplementary Figure 1: Original result of cell cycle analyses by flow cytometry for JQ1 treatment in H526, H69 and DMS79 cells.

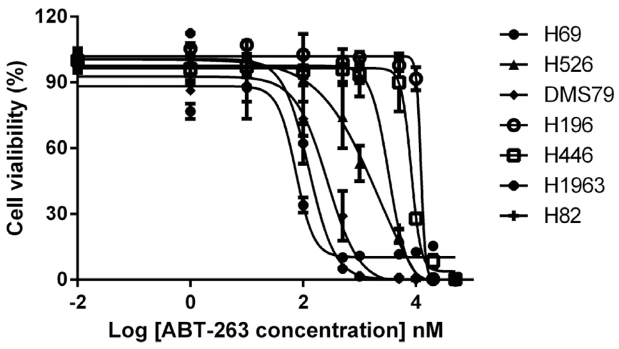

Table 2. The IC50 of ABT-263 in SCLC cell lines

Cell lines	H69	H526	DMS79	H196	H446	H1963	H82
IC50 (nM)	57.4	1930	301	6439	8180	124.8	3359

Supplementary Figure 2: Growth inhibition curves of ABT-263 in a panel of SCLC cell lines. SCLC cells were treated with different concentrations of ABT-263 for 72 hours. CellTiter-Glo Luminescent assay was performed to evaluate the cell proliferation. The IC50 values were determined from the sigmoidal dose–response curves using PRISM4 software.

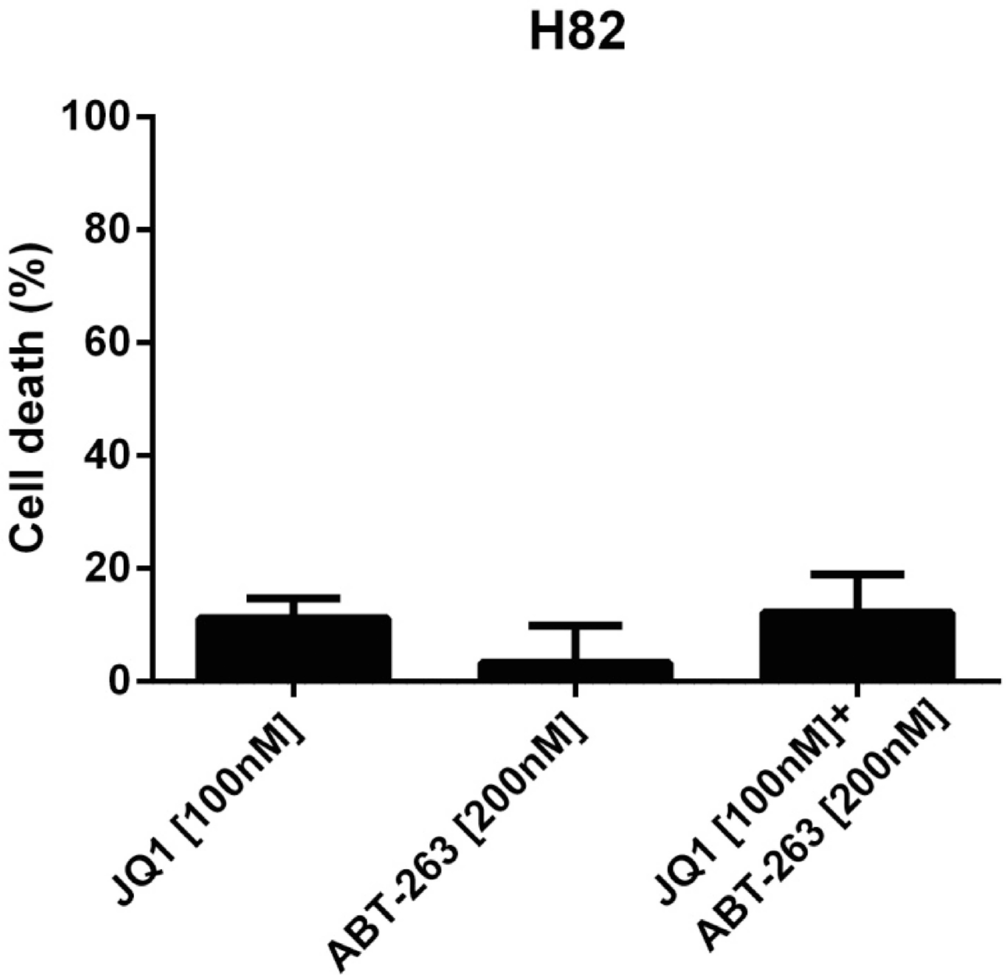

Supplementary Figure 3: Combination of JQ1 and ABT-263 did not induce more growth inhibition in H82 cells. H82 cells were treated with DMSO control, JQ1, ABT-263 or the combination of JQ1 and ABT-263 for 72 hours. After treatment, growth inhibition was determined by CellTiter-Glo Luminescent assay.

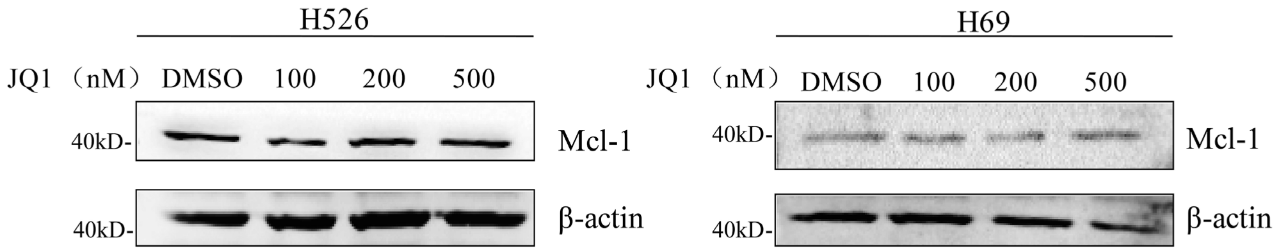

Supplementary Figure 4: JQ1 treatment has no impact on the expression of Mcl-1. H526 and H69 cells were treated with DMSO or JQ1 (100nM) for 24 hours, and then western blotting was performed to detect the expression levels of Mcl-1. *β*-Actin was used as a loading control.

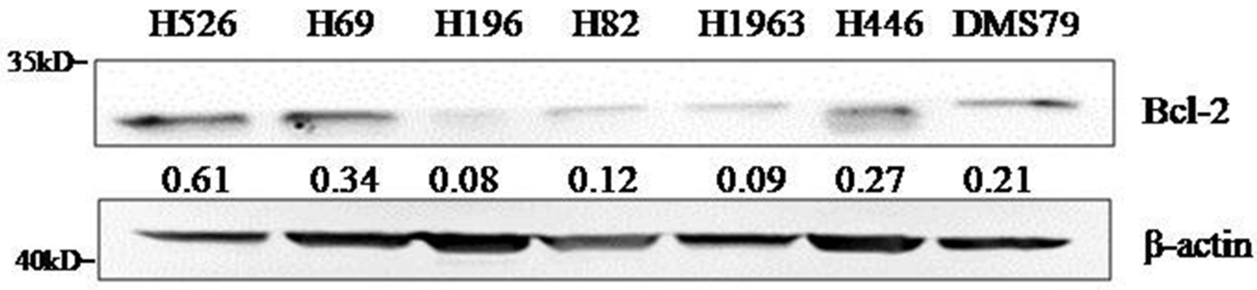

Supplementary Figure 5: Protein expression of Bcl-2 in a panel of SCLC cell lines by western blotting analysis. β-Actin was used as a loading control. Protein band intensities were quantified by ImageJ and normalized to Actin.
